# C-type natriuretic peptide-modified lipid vesicles: fabrication and use for the treatment of brain glioma

**DOI:** 10.18632/oncotarget.16641

**Published:** 2017-03-29

**Authors:** Jia-Shuan Wu, Li-Min Mu, Ying-Zi Bu, Lei Liu, Yan Yan, Ying-Jie Hu, Jing Bai, Jing-Ying Zhang, Weiyue Lu, Wan-Liang Lu

**Affiliations:** ^1^ State Key Laboratory of Natural and Biomimetic Drugs, Beijing Key Laboratory of Molecular Pharmaceutics and New Drug System, School of Pharmaceutical Sciences, Peking University, Beijing 100191, China

**Keywords:** C-type natriuretic peptide, lipid vesicles, BBB, brain glioma, neovasculatures

## Abstract

Chemotherapy of brain glioma faces a major obstacle owing to the inability of drug transport across the blood-brain barrier (BBB). Besides, neovasculatures in brain glioma site result in a rapid infiltration, making complete surgical removal virtually impossible. Herein, we reported a novel kind of C-type natriuretic peptide (CNP) modified vinorelbine lipid vesicles for transferring drug across the BBB, and for treating brain glioma along with disrupting neovasculatures. The studies were performed on brain glioma U87-MG cells *in vitro* and on glioma-bearing nude mice *in vivo*. The results showed that the CNP-modified vinorelbine lipid vesicles could transport vinorelbine across the BBB, kill the brain glioma, and destroy neovasculatures effectively. The above mechanisms could be associated with the following aspects, namely, long circulation in the blood; drug transport across the BBB *via* natriuretic peptide receptor B (NPRB)-mediated transcytosis; elimination of brain glioma cells and disruption of neovasculatures by targeting uptake and cytotoxic injury. Besides, CNP-modified vinorelbine lipid vesicles could induce apoptosis of the glioma cells. The mechanisms could be related to the activations of caspase 8, caspase 3, p53, and reactive oxygen species (ROS), and inhibition of survivin. Hence, CNP-modified lipid vesicles could be used as a carrier material for treating brain glioma and disabling glioma neovasculatures.

## INTRODUCTION

Brain glioma is a type of tumor that originates in the brain [1, 2]. Brain glioma cells infiltrate rapidly and disrupt the architecture of brain tissue, making complete resection virtually impossible [3]. Therefore, malignant brain glioma can rarely be cured by surgery or radiotherapy alone [4]. Accordingly, chemotherapy is used to clear tumor cells. However, treatment with anticancer drugs is hindered by the blood–brain barrier (BBB), which hinders the intravenously administered anticancer agents entering the region of brain glioma [5, 6]. Studies have shown that the BBB prevents uptake of all large-molecule and >98% of small-molecule drugs such as anticancer drugs, apart from temozolomide [7, 8]. In addition, the neovasculatures of brain glioma cannot be removed readily by resection, and angiogenesis further facilitates the growth of brain glioma [9, 10]. Therefore, how to effectively deliver anticancer drug to the region of brain glioma, and to disrupt the neovasculatures still remained to be unsolved issues.

Herein, we propose that a novel type of nanocarrier, C-type natriuretic peptide-modified lipid vesicles, can be used to transport anticancer drugs across the BBB, and then eliminate brain glioma and destroy neovasculatures. To construct these vesicles, C-type natriuretic peptide is conjugated with a vitamin E-derived conjugate, i.e., 22-amino acid D-α-tocopheryl polyethylene glycol_1000_ succinate (TPGS_1000_), as a ligand material, and modified onto lipid vesicles comprising phosphatidylcholine, cholesterol, and distearoylphosphatidylethanolamine polyethylene glycol_2000_ (DSPE-PEG_2000_). Vinorelbine is loaded into the vesicles as an anticancer agent.

The BBB is a physical (but also a changeable) barrier that maintains the homeostasis between the central nervous system and external environment [11]. It consists of brain microvascular endothelial cells (BMVECs), astrocyte foot process ensheathing vessels, and tight junctions, including zonula occludens-1 (ZO-1), claudin 5, and occludin [12]. The BBB is a selective barrier because some physiologic substances can transfer across the barrier by unique pathways, such as receptor-mediated transcytosis (RMT) [13], adsorptive transcytosis, and opening of tight junctions [14, 15].

CNP is a type of natriuretic peptide secreted from the heart [16], and can bind specifically to its receptor: natriuretic peptide receptor B (NPRB) [17]. NPRB has been reported to be expressed in the brain and blood vessels [18], and overexpressed in tumor cells such as pituitary adenomas [19]. Recent studies have shown that CNP can enhance BBB permeability by disrupting the tight-junction protein ZO-1 [20], and to attenuate angiogenesis [21]. TPGS_1000_ is a vitamin-E derivative that has been used as a functional material for enhancing cellular uptake [22, 23]. In the present study, CNP was conjugated with TPGS_1000_ for insertion onto the surface of lipid vesicles. We aimed to: (i) transport a drug across the BBB by RMT and opening of tight-junction; (ii) target brain glioma cells and neovasculatures.

DSPE-PEG_2000_ has also been used as a functional material for maintaining the stability and long circulation of drug carriers in blood. This is because DSPE-PEG_2000_ can avoid the rapid clearance of nanoparticles by the reticuloendothelial system (RES) [24], and facilitate accumulation of anticancer agents into tumor regions through the enhanced permeability and retention (EPR) effect [25].

Vinorelbine has a broad spectrum of anticancer activity, such as breast cancer and non-small-cell lung cancer [26]. It can eliminate cancer cells through interaction with tubulin, thereby leading to the mitotic arrest of cancer cells [27]. Moreover, vinorelbine has also shown the ability to destroy neovasculatures [28]. In the present study, vinorelbine was selected as a model anticancer agent and encapsulated in lipid vesicles for the treatment of brain glioma cells and their neovasculatures.

The objectives of the present study were to: (i) develop CNP-modified lipid vesicles loaded with vinorelbine; (ii) evaluate the effects on transportation of vinorelbine across the BBB; (iii) evaluate the effects on eliminating brain glioma cells and their neovasculatures; and (iv) explore the relevant mechanisms.

## RESULTS

### Synthesis of a CNP-TPGS_1000_ conjugate

**Figure 1:**
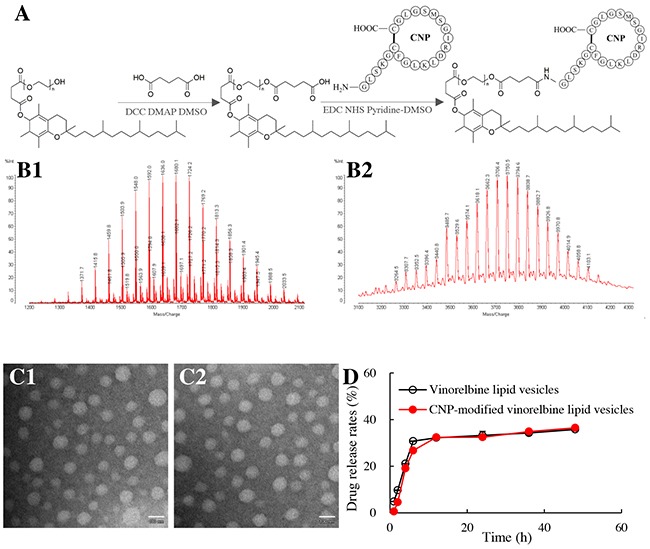
Synthesis of a CNP-TPGS1000 conjugate and characterization of CNP-modified lipid vesicles Notes: **(A)** synthetic route of CNP-TPGS_1000_. **(B1)** MALDI-TOF-MS spectrum of COOH-TPGS_1000_; **(B2)** MALDI-TOF-MS spectrum of CNP-TPGS_1000_. **(C1)** TEM image of vinorelbine lipid vesicles (Bar = 100 nm); **(C2)** TEM image of CNP-modified vinorelbine lipid vesicles (Bar = 100 nm). **(D)** release rates of vinorelbine. Data are the mean ± standard deviation (n=3).

To synthesize the CNP-TPGS_1000_ conjugate, TPGS_1000_ was reacted with glutaric acid to form COOH-TPGS_1000_. Then, the amino terminal of CNP was conjugated to the carboxyl terminal of COOH-TPGS_1000_ *via* a nucleophilic substitution reaction (Figure 1A). Thus, the final product, CNP-TPGS_1000_, was obtained.

Matrix-assisted laser desorption/ionization–time of flight–mass spectrometry (MALDI-TOF-MS) (Figure 1B) showed the mean mass of COOH-TPGS_1000_ to be *m/z* 1636 (Figure 1B1). The difference in mass between TPGS_1000_ and COOH-TPGS_1000_ was equal to the mass of glutaric acid and H_2_O, indicating the successful synthesis of COOH-TPGS_1000_.

The mean mass of CNP-TPGS_1000_ was *m/z* 3816 (Figure 1B2). The difference in mass between CNP-TPGS_1000_ and COOH-TPGS_1000_ was equal to the mass of CNP and H_2_O, thereby demonstrating the successful synthesis of CNP-TPGS_1000_.

### CNP-modified vinorelbine lipid vesicles

CNP-modified vinorelbine lipid vesicles were round according to transmission electron microscopy (TEM; Figure 1C1, 1C2). Their mean diameter was 106.7 ± 1.0 nm, and they had a narrow polydispersity index (0.23). The zeta potential of CNP-modified vinorelbine lipid vesicles was −6.48±0.21 mV. The encapsulation efficiency (EE) of vinorelbine within the vesicles was >90% (Table 1). Release of vinorelbine from the lipid vesicles *in vitro* was <10% in the initial 2 h, and <40% over 48 h in the blood components-containing buffer (Figure 1D).

**Table 1:** Characterization of lipid vesicles

Formulations	Encapsulation efficiency (%)	Particle size (nm)	PDI	Zeta potential (mV)
CNP-modified vinorelbine lipid vesicles	93.34±0.66	106.67±1.03	0.237±0.008	-6.48±0.21
Vinorelbine lipid vesicles	97.32±1.46	104.03±1.08	0.232±0.008	-7.32±0.22
Blank CNP-modified lipid vesicles	-	100.77±0.65	0.063±0.006	-9.37±0.12
Blank lipid vesicles	-	96.31±1.13	0.168±0.010	-6.37±0.41

Notes: PDI, polydispersity index; data are the mean ± standard deviation (n = 3).

Control formulations, vinorelbine lipid vesicles, and blank CNP-modified lipid vesicles were characterized and had similar properties.

### Transport across the BBB

**Figure 2:**
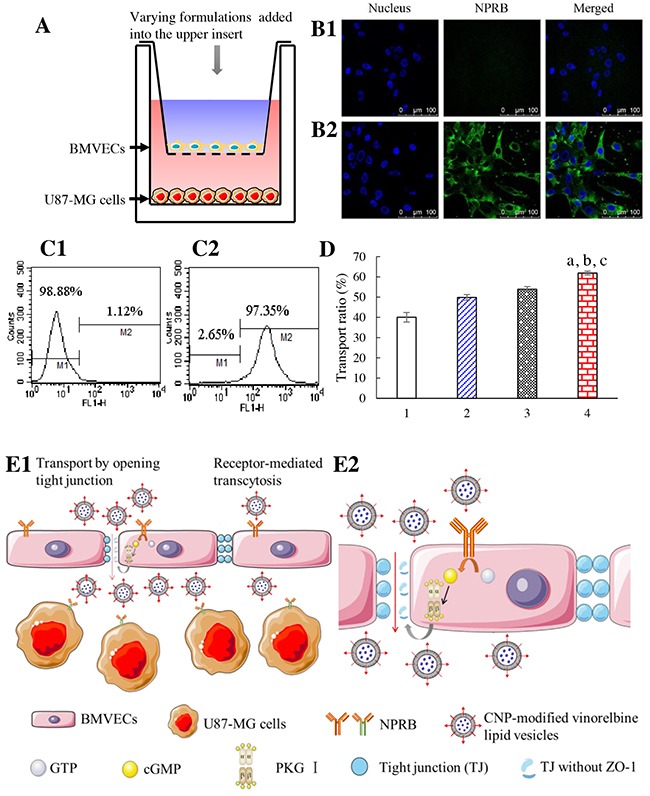
Transport across the co-cultured BBB Notes: **(A)** *in vitro* co-culture BBB model. **(B)** NPRB expression in BMVECs is identified by confocal laser scanning microscopy; **(B1)** isotope control; **(B2)** anti-NPRB antibody. **(C)** NPRB expression in BMVECs is identified by flow cytometry; **(C1)** isotope control; **(C2)** anti-NPRB antibody. **(D)** transport across the BBB followed by killing of glioma cells; 1, free vinorelbine; 2, vinorelbine lipid vesicles; 3, CNP-modified vinorelbine lipid vesicles pretreated with Rp-8-CPT-cGMPS; 4, CNP-modified vinorelbine lipid vesicles. p < 0.05, a, *vs*. 1; b, *vs*. 2; c, *vs*. 3. Data are the mean ± standard deviation (n = 3). **(E)** CNP-modified vinorelbine lipid vesicles transporting across the BBB (schematic); E1, normal view; E2, enlarged view.

To evaluate the transport effect across the BBB, a co-culture BBB model was established with BMVECs seeded on the upper insert whereas brain glioma (U87-MG) cells were seeded on the lower well (Figure 2A). Values for the transendothelial electrical resistance between the upper insert and lower well were >300 Ωcm^2^, which resulted in well-organized tight junctions between BMVECs, thereby confirming successful establishment of the BBB co-culture model.

NPRB expression in BMVECs was identified by flow cytometry and confocal laser scanning microscopy (CLSM; Figure 2B, 2C). NPRB was labeled with anti-NPRB antibody and shown as green fluorescent structures (Figure 2B2). NPRB was not detected in the control group with isotype antibody (Figure 2B1). NPRB expression in BMVECs was 97.35% (Figure 2C2), which was higher than that of the isotype control (Figure 2C1).

After application of a drug formulation into the upper insert, the transport ability across the BBB model was characterized by the inhibitory effects upon U87-MG cells in the lower well (Figure 2D). After treatment with various formulations, the ranking of the transport ratio was CNP-modified vinorelbine lipid vesicles (61.88±0.94%) > CNP-modified vinorelbine lipid vesicles incubated with a cyclic guanosine monophosphate (cGMP) inhibitor (Rp-8-CPT-cGMPS; 46.10±1.28%) > vinorelbine lipid vesicles (49.84±1.41%) > free vinorelbine (40.05±2.37%).

Transport results showed that CNP-modified vinorelbine lipid vesicles were transferred across the BBB model through NPRB-mediated transcytosis by specific binding to NPRB expressed in BMVECs (Figure 2E1). After application of a cGMP inhibitor (Rp-8-CPT-cGMP), the transport ability of CNP-modified vinorelbine lipid vesicles was decreased significantly. This finding suggested that CNP-modified vinorelbine lipid vesicles could transfer across the BBB by disruption of tight junctions as well (Figure 2E2) [20].

### Targeting uptake and toxicity to brain glioma cells

**Figure 3:**
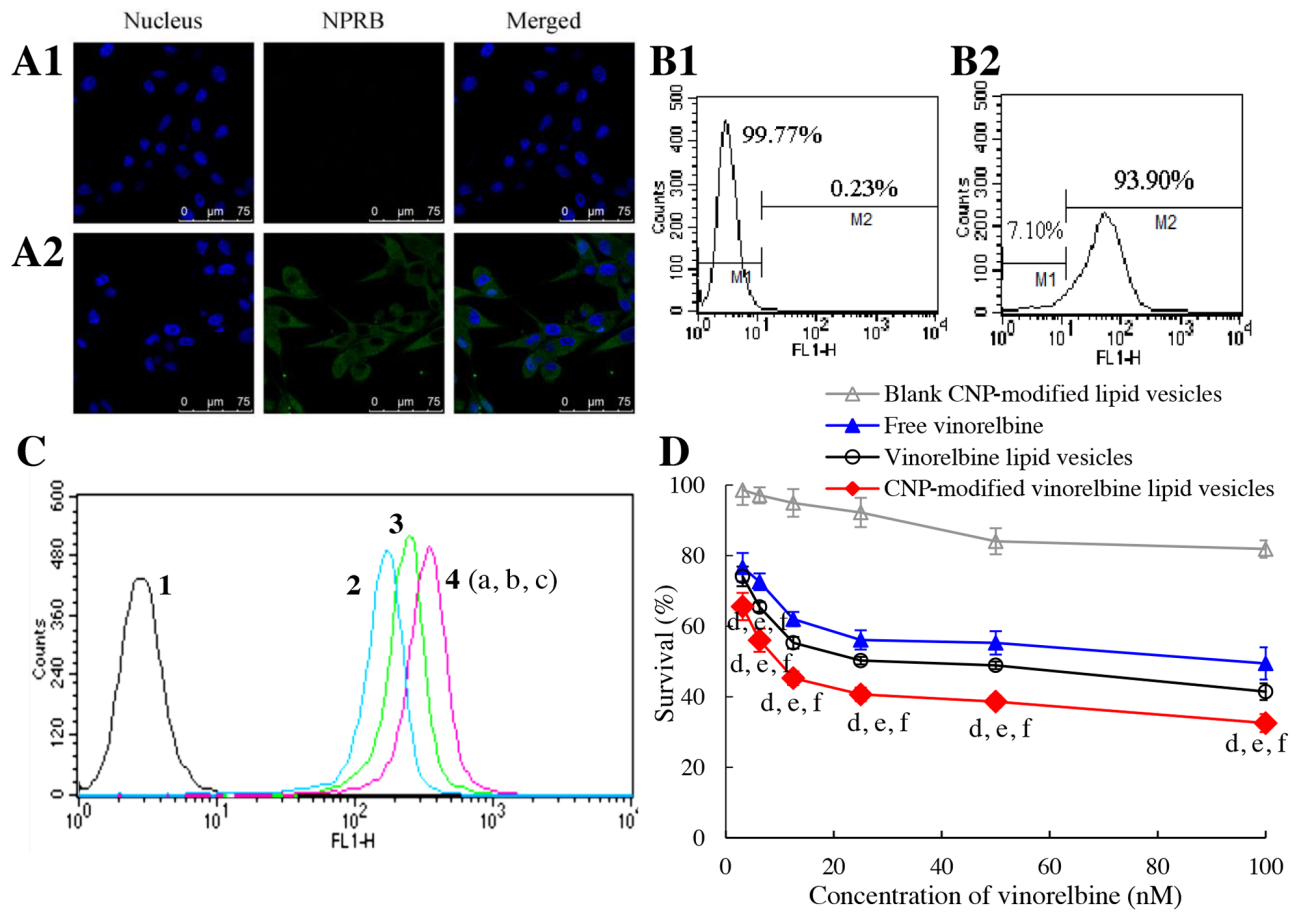
Targeting uptake by brain glioma cells and cytotoxicity Notes: **(A)** NPRB expression in U87-MG cells identified by confocal laser scanning microscopy; **(A1)** isotope control; **(A2)** anti-NPRB antibody. **(B)** NPRB expression in U87-MG cells identified by flow cytometry; **(B1)** isotope control; **(B2)** anti-NPRB antibody. **(C)** cellular uptake in U87-MG cells; 1, blank control; 2, free coumarin; 3, coumarin lipid vesicles; 4, CNP-modified coumarin lipid vesicles. p < 0.05, a, *vs*. 1; b, *vs*. 2; c, *vs*. 3. Data are the mean ± standard deviation (n = 3). **(D)** toxicity to U87-MG cells. p < 0.05, d, *vs*. blank control; e, vs. free vinorelbine; f, vs. vinorelbine lipid vesicles. Data are the mean ± standard deviation (n = 6).

To evaluate the targeting effect of CNP-modified lipid vesicles on brain glioma cells, NPRB expression in U87-MG cells was identified by flow cytometry and CLSM (Figure 3A, 3B), respectively. NPRB was labeled with anti-NPRB antibody and shown as green fluorescent structures (Figure 3A2). NPRB was not detected in the control group with isotype antibody (Figure 3A1). NPRB expression in U87-MG cells was 93.90% (Figure 3B2), which was higher than that for the isotype control (Figure 3B1).

To evaluate uptake by U87-MG cells, coumarin-labeled lipid vesicles were prepared and used as an indicator of green fluorescence. Cellular uptake was measured by flow cytometry (Figure 3C). The ranking of cellular uptake was CNP-modified coumarin lipid vesicles (2.98±0.03) > coumarin lipid vesicles (2.18±0.07) > free coumarin (which was given a value of 1 as a reference).

To assess cytotoxicity, the inhibitory effects upon U87-MG cells were evaluated by a sulforhodamine B (SRB) staining assay. After treatment with various drug formulations, the ranking of toxicity to U87-MG cells was CNP-modified vinorelbine lipid vesicles > vinorelbine lipid vesicles > free vinorelbine > blank CNP-modified vinorelbine lipid vesicles (Figure 3D).

### Apoptosis of glioma cells and signaling pathways

**Figure 4:**
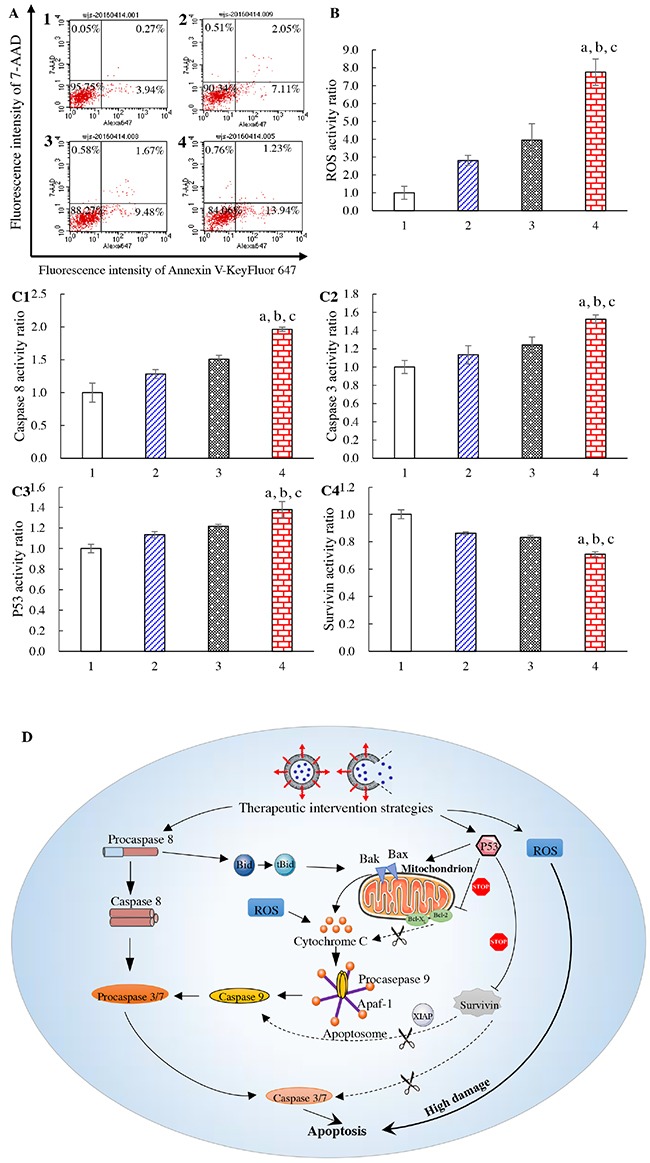
Apoptosis of brain glioma cells and mechanism of action Notes: **(A)** induction of apoptotic effects on U87-MG cells. **(B)** ROS activity of U87-MG cells. **(C1)** activity of caspase 8; **(C2)** activity of caspase 3; **(C3)** activity of p53; **(C4)** activity of survivin. **(D)** mechanism of apoptosis in U87-MG cells (schematic). 1, blank control; 2, free vinorelbine; 3, vinorelbine lipid vesicles; 4, CNP-modified vinorelbine lipid vesicles. p < 0.05, a, *vs*. 1; b, *vs*. 2; c, *vs*. 3. Data are the mean ± standard deviation (n = 4).

Apoptosis of U87-MG cells was evaluated by flow cytometry (Figure 4A). After incubation with blank medium, free vinorelbine, vinorelbine lipid vesicles, or CNP-modified vinorelbine lipid vesicles, the total percentage of apoptosis in U87-MG cells was 4.36±0.19%, 10.99±1.37%, 12.80±1.53%, and 16.70±1.06%, respectively.

Release of reactive oxygen species (ROS) in U87-MG cells induced by drug treatment was also evaluated by flow cytometry (Figure 4B). After addition of blank medium, free vinorelbine, vinorelbine lipid vesicles, or CNP-modified vinorelbine lipid vesicles, ROS activity in glioma cells was 1.00±0.37, 2.81±0.78, 3.95±0.92, and 7.76±0.74, respectively.

To evaluate apoptotic signaling pathways, quantification of apoptosis-related enzymes and proteins in U87-MG cells was evaluated by a high-content analysis system (Figure 4C). After incubation with blank medium, free vinorelbine, vinorelbine lipid vesicles, or CNP-modified vinorelbine lipid vesicles, the activity of the upstream apoptotic enzyme caspase 8 was 1.00±0.15, 1.28±0.07, 1.50±0.06, and 1.96±0.03, respectively (Figure 4C1). The activity of the downstream apoptotic enzyme caspase 3 was 1.00±0.07, 1.13±0.10, 1.24±0.09, and 1.52±0.05, respectively (Figure 4C2). The activity of the apoptosis protein p53 was 1.00±0.04, 1.13±0.03, 1.22±0.02, and 1.38±0.08, respectively (Figure 4C3). The activity of the apoptosis-suppressing protein survivin was 1.00±0.03, 0.86±0.01, 0.83±0.01, and 0.71±0.02, respectively (Figure 4C4). Compared with control formulations, CNP-modified vinorelbine lipid vesicles increased the activity of caspase 8, caspase 3, and p53 significantly, but reduced the activity of survivin.

Based on the measurements of important apoptotic proteins in U87-MG cells, apoptotic signaling pathways are explained using a schematic representation in Figure 4D.

### Targeting and disruption of glioma neovasculatures *in vitro*

**Figure 5:**
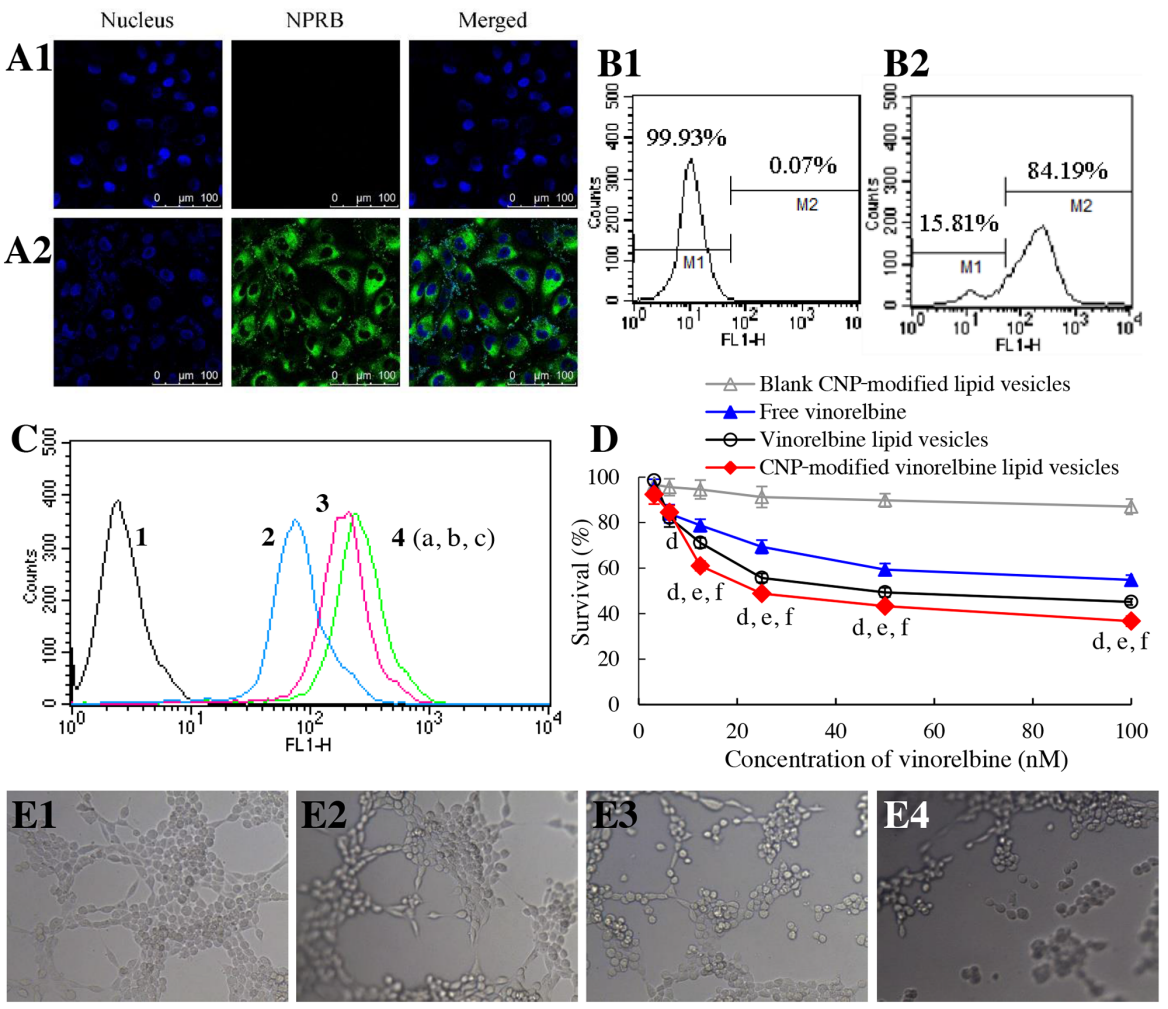
Targeting and disruption of glioma neovasculatures *in vitro* Notes: **(A)** NPRB expression in HUVECs identified by confocal laser scanning microscopy; **(A1)** isotope control; **(A2)** anti-NPRB antibody. **(B)** NPRB expression in HUVECs identified by flow cytometry; **(B1)** isotope control; **(B2)** anti-NPRB antibody. **(C)** cellular uptake in HUVECs; 1, blank control; 2, free coumarin; 3, coumarin lipid vesicles; 4, CNP-modified coumarin lipid vesicles. p < 0.05, a, *vs*. 1; b, *vs*. 2; c, *vs*. 3. Data are the mean ± standard deviation (n = 3). **(D)** inhibitory effects on HUVECs. p < 0.05, d, *vs*. blank control; e, *vs*. free vinorelbine; f, vs. vinorelbine lipid vesicles. Data are the mean ± standard deviation (n = 6). **(E)** destruction of neovasculatures *in vitro* after treatment with various formulations; **(E1)** blank control; **(E2)** free vinorelbine; **(E3)** vinorelbine lipid vesicles; **(E4)** CNP-modified vinorelbine lipid vesicles.

To evaluate the targeting effect of CNP-modified lipid vesicles on human umbilical vein endothelial cells (HUVECs), NPRB expression in HUVECs was identified by flow cytometry and CLSM (Figure 5A, 5B). NPRB was labeled with anti-NPRB antibody and shown as green fluorescent structures (Figure 5A2). NPRB was not detected in the control group with isotype antibody (Figure 5A1). NPRB expression in HUVECs was 84.19% (Figure 5B2), which was higher than that for the isotype control (Figure 5B1).

To evaluate uptake by HUVECs, cellular uptake was measured by flow cytometry (Figure 5C). Results showed that the ranking of cellular uptake was CNP-modified coumarin lipid vesicles (3.06±0.15) > coumarin lipid vesicles (2.49±0.12) > free coumarin (using a value of 1 as a reference).

To assay cytotoxicity, the inhibitory effects upon HUVECs were evaluated by a SRB assay. After treatment with various drug formulations, the ranking of cytotoxicity to HUVECs was CNP-modified vinorelbine lipid vesicles > vinorelbine lipid vesicles > free vinorelbine > blank CNP-modified vinorelbine lipid vesicles (Figure 5D).

To evaluate the disruptive effect, model neovasculatures were built using HUVECs. These model neovasculatures showed loop-like structures (Figure 5E1). A significant number of these structures was destroyed by treatment with CNP-modified vinorelbine lipid vesicles (Figure 5E4) compared with those treated with control formulations (Figure 5E2, 5E3).

### Imaging in glioma-bearing mice *in vivo*

**Figure 6:**
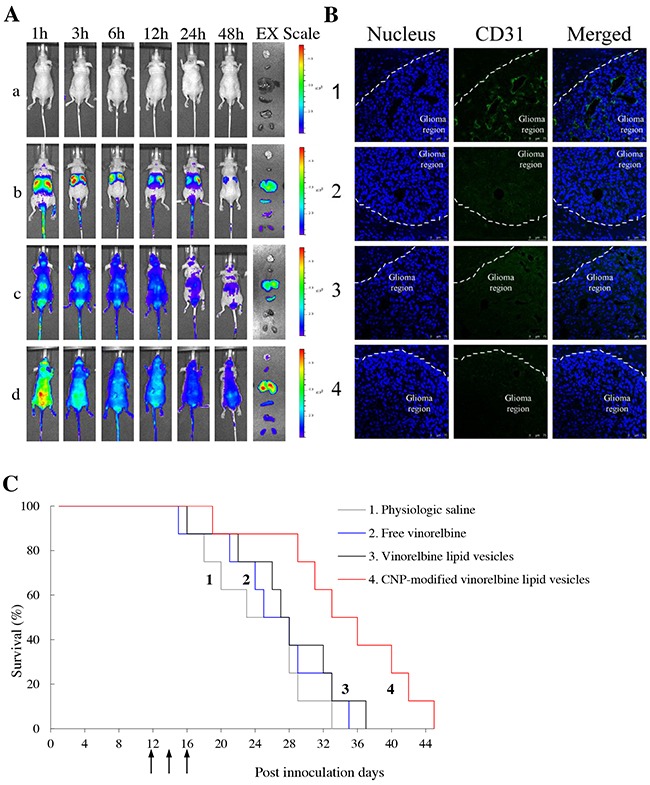
Real-time imaging and overall anticancer efficacy in glioma-bearing mice *in vivo* Notes: **(A)** *in vivo* real-time images and *ex vivo* images of glioma-bearing brains and organs at 48 h; a, physiologic saline; b, free DiR; c, DiR lipid vesicles; d, CNP-modified DiR lipid vesicles. **(B)** disruptive effects on brain glioma neovasculatures in glioma-bearing mice. Glioma neovasculatures are indicated as green fluorescence stained by anti-CD31 antibody. White lines indicate the boundary between the brain-glioma region and normal brain tissue. **(C)** Kaplan–Meier survival curves of glioma-bearing mice treated with various formulations (n = 8). 1, physiologic saline; 2, free vinorelbine; 3, vinorelbine lipid vesicles; 4, CNP-modified vinorelbine lipid vesicles.

Real-time imaging of fluorescent probe DiIC_18_(7) (1,1’-Dioctadecyl-3,3,3’,3’- tetramethylindotricarbocyanine iodide) (DiR)-labeled formulations was undertaken on brain glioma-bearing mice (Figure 6A). After injection of various fluorescent probe DiR-labeled formulations, CNP-modified DiR lipid vesicles indicated the strongest fluorescence signal in the brain.

In the sacrificed-mice group, the brain, heart, liver, spleen, lungs and kidneys were excised for examination *ex vivo* 48 h after injection. Among various formulations, a strong fluorescence signal of CNP-modified DiR lipid vesicles was observed in brain tissue. In comparison, the fluorescence signals of other formulations at 48 h were weakly visible in brain tissue but were distributed mainly in the liver and spleen.

### Disruption of neovasculatures and anticancer efficacy in brain glioma-bearing mice

To evaluate the disruptive effect on neovasculatures in vivo, brain tissues were excised from mice killed at 18 day after multiple intravenous administrations of physiologic (0.9%) saline, free vinorelbine lipid vesicles, or CNP-modified vinorelbine lipid vesicles on days 12, 14 and 16. Tissues were frozen, sliced, and stained with CD31 for observation (Figure 6B). Brain nuclei were identified as blue fluorescent structures, whereas neovasculatures were green. After treatment with CNP-modified vinorelbine lipid vesicles, a significant number of neovasculatures in the brain-glioma region were destroyed compared with those treated with control formulations.

To evaluate anticancer efficacy in brain glioma-bearing mice, Kaplan–Meier survival curves were used. After treatment with physiologic saline, free vinorelbine, vinorelbine lipid vesicles, or CNP-modified vinorelbine lipid vesicles, mean survival time was 24.38, 26.25, 27.63 and 34.38 days, respectively. Survival of mice treated with CNP-modified vinorelbine lipid vesicles was extended significantly compared with those treated with physiologic saline (p = 0.008), free vinorelbine (p = 0.024) or vinorelbine lipid vesicles (p = 0.047) (Figure 6C).

In addition, the preliminary toxicity assessments on various formulations were evaluated by blood examination and by histopathological observation on major organs. As compared with physiological saline, there were no abnormalities observed in blood indicators (Supplementary Table 1), and in major organs (Supplementary Figure 1) after treatment with CNP-modified vinorelbine lipid vesicles.

## DISCUSSION

Brain glioma is the most common primary tumor of the central nervous system, with an annual incidence of ≈6 per 100,000 individuals [29]. It has a very aggressive clinical course, with a median survival of <17 months [30]. Treatment of brain glioma is a major challenge owing to the hindrance of the BBB to most anticancer agents, and the regeneration of residual glioma cells with angiogenic support in the tumor region [31]. We developed a new type of chemotherapy formulation that could be transported across the BBB to eliminate brain glioma cells and disrupt glioma neovasculatures.

In the present study, a newly synthesized material, CNP-TPGS_1000_, was inserted onto the surface of lipid vesicles for transportation of a drug across the BBB to target glioma cells and endothelial cells within neovasculatures in the glioma region. We were able to fabricate CNP-modified vinorelbine lipid vesicles that were round, of nano-scale size, and which showed low leakage and high encapsulation of vinorelbine (Table 1; Figure 1C, 1D). These properties allow lipid vesicles to be stable in blood and to facilitate drug accumulation in the brain-glioma region *via* an EPR effect.

Studies have shown that the CNP receptor, NPRB, is expressed in the brain and blood vessels, and overexpressed in pituitary adenomas. We demonstrated that NPRB is overexpressed in BMVECs, HUVECs, and U87-MG cells (Figure 2C, 5B, 3B). As a specific ligand, CNP can bind to NPRB and mediate the transport of substances across the BBB by opening of tight junctions [20]. Also, CNP can inhibit expression of vascular endothelial growth factor (VEGF) [21]. Based on a BBB model, the present study showed the existence of two mechanisms during transport of CNP-modified vinorelbine lipid vesicles across the BBB: RMT and opening of tight junctions.

RMT was evidenced by the fact that significant numbers of CNP-modified vinorelbine lipid vesicles could be transported across the BBB by targeting interaction with highly expressed NPRB on BMVECs in the BBB (Figure 2A–2E). This action occurred because RMT enables selective uptake of macromolecules across the BBB, with endothelial cells possessing receptors for the uptake of different ligands, including enzymes, proteins and growth factors [32].

Opening of tight junctions was observed by addition of an inhibitor of cGMP-dependent protein kinase: Rp-8-CPT-cGMPS. In the present study, CNP-modified vinorelbine lipid vesicles could open the tight junctions of the BBB *via* signaling through cGMP and protein kinase G [20]. When pretreated with Rp-8-CPT-cGMPS, the transport ability of CNP-modified vinorelbine lipid vesicles was decreased as compared with those not treated with Rp-8-CPT-cGMPS. Furthermore, in the presence of inhibitor Rp-8-CPT-cGMPS, the transport ability of CNP-modified vinorelbine lipid vesicles was still higher than that of vinorelbine lipid vesicles (Figure 2D). Tight junctions can be highly dynamic structures that provide a reversible membranous barrier for the BBB. Recent studies have demonstrated that modulators of tight junctions can also improve the permeability of drugs across the BBB *via* phosphorylation of tight-junction proteins, such as activation of signaling pathways for protein kinase A and G [33].

RMT was also responsible for the enhanced uptake in brain glioma cells so that CNP-modified vinorelbine lipid vesicles could kill significant numbers of U87-MG cells (Figure 3D). This was evidenced using a cellular uptake assay in which CNP-modified lipid vesicles could increase coumarin uptake in glioma cells through specific binding to highly expressed NPRB in U87-MG cells (Figure 3A–3C).

Induction of apoptosis by CNP-modified vinorelbine lipid vesicles contributed to the enhanced killing effect on brain glioma cells. The strongest apoptotic induction was observed after treatment with CNP-modified vinorelbine lipid vesicles through activation of the apoptotic enzymes caspase 8, caspase 3 and the apoptosis protein p53, or by inhibition of the apoptosis-suppressing protein survivin (Figure 4A, 4C). Furthermore, CNP-modified vinorelbine lipid vesicles could induce apoptosis significantly by a ROS pathway (Figure 4B). As shown in Figure 4D, three apoptotic signaling pathways were involved. First, activation of upstream caspase 8 was involved with a death receptor signaling pathway, followed by initiation of caspase 3, which led to apoptosis [34]. Second, when the apoptosis protein p53 was activated, it permeabilized mitochondrial membranes directly, causing the release of cytochrome C [35]. Then, the apoptosis protein p53 facilitated the binding of cytochrome C to apoptotic protease activating factor-1 while inhibiting the apoptosis-suppressing protein survivin. These actions activated the downstream effector caspase 9, which also caused accumulation of caspase 3 [36]. Finally, ROS participated in the release of cytochrome C and induced high levels of apoptosis directly. Such activation/inhibition resulted in a cascade of reactions that led to programmed death of the glioma cells involved in cellular stress [37].

HUVECs were chosen to develop a model for tumor neovasculatures [38, 39] and to evaluate the anti-angiogenic effect of various vinorelbine formulations. The strongest inhibitory activity upon HUVECs was in the group treated with CNP-modified vinorelbine lipid vesicles, and the mechanism could be explained by increasing cellular uptake through binding to highly expressed NPRB in HUVECs (Figure 5D). Furthermore, the microscope images from the model of glioma neovasculatures established with HUVECs in three-dimensional Matrigel® demonstrated that the most significant disrupting effect on neovasculatures was after treatment with CNP-modified vinorelbine lipid vesicles, and resulted from increased cellular uptake *via* specific binding with NPRB (Figure 5C, 5E).

Recent studies have demonstrated the eliminating effects on neovasculatures of vinorelbine *in vitro* and *in vivo*. Vinorelbine could destruct neovasculatures *in vitro*, by suppressing pro-angiogenic proteins IL-8 an COX-2, by increasing anti-angiogenic proteins PPARγ and CD36 [40], and by attenuating SDF-1/CXCR4 pathway involved in the proliferation and migration of neovasculatures [41]. Besides, vinorelbine could destroy the neovasculatures *in vivo* by descreasing pro-angiogenic proteins IL-8 and FGF2 [42].

The interaction between CNP ligand and NPRB receptor may also have influence on eliminating neovasculatures. It was reported that the interaction could attenuate angiogenesis by inhibiting VEGF [21, 43]. The exact mechanism awaits further investigation. Based on the present study, CNP-modified vinorelbine lipid vesicles demonstrate an evident capability to disrupt glioma neovasculatures *in vitro* and *in vivo*. The mechanism was associated with the fact that CNP mediated the targeting of CNP-modified vinorelbine lipid vesicles to glioma neovasculatures, thereby enhancing the uptake of vinorelbine, and the destroying effect of vinorelbine on neovasculatures.

Experiments were conducted in intracranial glioma-bearing nude mice *in vivo*, including imaging of drug distribution and evaluation of overall anticancer efficacy. Imaging *in vivo* and *ex vivo* using the fluorescent dye DiR demonstrated that CNP-modified vinorelbine lipid vesicles had a longer circulatory effect and could accumulate more in brain-glioma regions compared with other formulations (Figure 6A). In contrast, free DiR and DiR lipid vesicles were distributed mainly in the liver and spleen, and were cleared rapidly in the blood circulation.

A CD31 immunofluorescence staining method and survival curves were used to evaluate the destructive effects on glioma neovasculatures and overall anticancer effects. CNP-modified vinorelbine lipid vesicles could disrupt glioma neovasculatures effectively and exhibited the strongest anticancer effect in glioma-bearing mice (Figure 6B, 6C). This phenomenon could be explained by the enhanced transport of vinorelbine across the BBB and increased uptake in glioma cells and glioma neovasculatures.

## MATERIALS AND METHODS

### Materials and cells

CNP was synthesized by Bankpeptide (Hefei, China). Vinorelbine was purchased from Nanjing Tianzun Zezhong Chemicals (Nanjing, China). Egg phosphatidylcholine (EPC) was purchased from Lipoid (Ludwigshafen, Germany). DSPE-PEG_2000_ was obtained from the NOF Corporation (Tokyo, Japan). Cholesterol, N-hydroxysuccinimide (NHS), EDC and glutaric acid were purchased from J&K Scientific (Beijing, China). TPGS_1000_, dicyclohexylcarbodiimide (DCC) and 4-dimethylaminopyridine (DMAP) were purchased from Sigma–Aldrich (Saint Louis, MO, USA). Dimethyl sulfoxide (DMSO) was obtained from Amresco (Solon, OH, USA). All other reagents were purchased from Beijing Chemical Reagents (Beijing, China).

Human brain glioma (U87-MG) cells and HUVECs were purchased from the Institute of Basic Medical Science, Chinese Academy of Medical Science (Beijing, China). Murine BMVECs were donated by the Institute of Clinical Medical Sciences at China–Japan Friendship Hospital (Beijing, China). U87-MG cells were cultured in Eagle's minimum essential medium supplemented with 1% non-essential amino acids and 10% fetal bovine serum (FBS; Gibco, Carlsbad, CA, USA). HUVECs were cultured in Dulbecco's modified Eagle's medium (DMEM) supplemented with 10% FBS. BMVECs were cultured in DMEM containing 20% FBS, 100 mg/mL endothelial cell growth factor, and 40 U/mL heparin. Male BALB/C nude mice (18–22 g) were obtained from the Experimental Animal Center of Peking University (Beijing, China). All cells were cultured in a humidified atmosphere of 5% CO_2_ at 37°C. All culture media and growth factors were purchased from Macgene Biotech (Beijing, China) unless stated otherwise. Male BALB/C nude mice (18–22 g) were obtained from the Experimental Animal Center of Peking University.

Experiments involving human cells and mice were approved by the Ethics Committee of the Health Science Center of Peking University.

### Synthesis of a CNP-TPGS_1000_ conjugate

A CNP-conjugate was synthesized. Briefly, glutaric acid (0.2 mmol), DMAP (0.1 mmol) and DCC (0.24 mmol) were dissolved in DMSO (2 mL). The mixture was stirred using a magnetic stirrer for 2 h at room temperature. TPGS_1000_ (0.04 mmol) was added to the mixture, which was stirred in a light-resistant container for 24 h at room temperature. Subsequently the crude product was transferred into regenerated cellulose dialysis tubing (molecular weight cutoff, MWCO, 1500) and dialyzed against deionized water for 48 h to remove uncoupled reagents. The resultant COOH-TPGS_1000_ was obtained by freeze-drying [44].

Afterwards, COOH-TPGS_1000_ (10 mmol), EDC (40 mmol) and NHS (70 mmol) were dissolved in pyridine-DMSO (1:1, *v/v*, 2 mL). The mixture was stirred for 30 min, followed by addition of CNP (Gly-Leu-Ser-Lys-Gly-Cys-Phe-Gly-Leu-Lys-Leu-Asp-Arg-Ile-Gly-Ser-Met-Ser-Gly-Leu-Gly-Cys; disulfide bridge: Cys6 - Cys22; 10 mmol), and stirring for 24 h. The crude product was dialyzed against deionized water in regenerated cellulose dialysis tubing (MWCO, 3000) for 48 h to remove unreacted raw materials and solvents. The resultant product was freeze-dried and CNP-TPGS_1000_ was obtained. The product was confirmed and characterized by MALDI-TOF-MS using a Shimadzu (Tokyo, Japan) system.

### CNP-modified vinorelbine lipid vesicles

Blank CNP-modified lipid vesicles were prepared as described in our previous report [45]. The constituents, EPC, cholesterol, DSPE-PEG_2000_ and CNP-TPGS_1000_ (60:30:5:5; mol/mol), were mixed in dichloromethane in a pear-shaped bottle. Blank lipid vesicles were prepared using TPGS_1000_ to replace CNP-TPGS_1000_. The solvent was removed by the rotary-film evaporation method at 40°C. The lipid film was hydrated with 250 mM ammonium sulfate *via* sonication in a water bath for 5 min. The crude product was treated using an ultrasonic cell disruptor (Scientz Biotechnology, Ningbo, China) for 10 min and lipid vesicles were extruded thrice through polycarbonate membranes (pore size = 200 nm; Millipore, Bedford, MA, USA).

For preparation of CNP-modified vinorelbine lipid vesicles, the blank lipid vesicles described above were dialyzed (MWCO, 12,000–14,000 Da) thrice in HEPES-buffered saline (25 mM HEPES, 150 mM NaCl) for 24 h and incubated in vinorelbine solution at 40°C with intermittent shaking for 30 min (lipids:drug = 20:1, *w/w*). The other two types of lipid vesicles, coumarin-labeled CNP-modified lipid vesicles (lipids:coumarin = 500:1, *w/w*) and DiR-labeled lipid vesicles (lipids:DiR = 200:1, *w/w*), were prepared using similar procedures.

The particle size, polydisperity index, and zeta potential were measured by a Nano Series Zen 4003 Zetasizer (Malvern, Malvern, UK). A transmission electron microscope (Tecnai G2 20ST; FEI, Tokyo, Japan) was used to observe the morphology of lipid vesicles.

The EE (%) of vinorelbine was calculated using the formula: EE = (W_e_/W_total_) × 100%, where W_e_ is the measured amount of vinorelbine in lipid vesicle suspensions after passing over a Sephadex G-50 column, and W_total_ is the total amount of vinorelbine in an equal volume of initial lipid vesicle suspensions. The release rate (RR, %) *in vitro* of vinorelbine from lipid vesicles was calculated using the formula: RR = (W_i_/W_total_) × 100%, where W_i_ is the measured amount of vinorelbine at the i^th^ time-point in the release medium phosphate-buffered saline (PBS, pH 7.4, 137 mM NaCl, 2.7 mM KCl, 8 mM Na_2_HPO_4_ and 2 mM KH_2_PO_4_) containing 10% fetal bovine serum, and W_total_ is the total amount of vinorelbine in an equal volume of lipid vesicle suspensions before dialyses. Methanol was used to disrupt lipid vesicles, and the concentration of vinorelbine was analyzed using high-performance liquid chromatography (HPLC; Shimadzu). HPLC analyses were carried out using an ODS column (Nucleodur 100–5C_18_ column, 250 mm × 4.6 mm, 5.0 mm; Macherey-Nagel, Easton, PN, USA) at 268 nm and 40°C. The mobile phase consisted of acetonitrile, 0.05 M KH_2_PO_4_ and triethylamine (pH = 4.0; 34:66: 0.3, *v/v*). The flow rate was 1.0 mL/min. Each assay was repeated in triplicate.

### NPRB staining

A flow cytometer (Becton Dickinson, San Jose, CA, USA) was used to observe expression of NPRB (the receptor of the CNP ligand) in U87-MG cells, HUVECs and BMVECs. Cells were seeded in a 12-well culture plate at 1.5×10^5^ cells/well in 2 mL of growth medium and cultured at 37°C. Cells were fixed with methanol (80%, 5 min), and incubated in PBS (pH 7.4) containing 10% normal goat serum and 0.3 M glycine to block non-specific protein–protein interactions. Then, cells were incubated with anti-NPRB antibody (1 μg/1×10^6^ cells; Abcam, Cambridge, UK) for 30 min at 22°C, whereas control experiments were undertaken using isotype antibody (2 μg/1×10^6^ cells; Abcam). The secondary antibody was goat anti-rabbit IgG (1/500 dilution, 30 min) at 22°C. After incubation, NPRB expression was measured using a flow cytometer with 1×10^4^ events collected, and expression was denoted by the fluorescence intensity [46].

A confocal laser scanning fluorescence microscope with built-in software (Leica, Heidelberg, Germany) was used to further observe NPRB expression in U87-MG cells, HUVECs and BMVECs. Briefly, cells were seeded into chambered coverslips at 1.5×10^5^ cells/well. After incubation for 24 h, cells were fixed using paraformaldehyde (4%, 10 min) and then incubated in PBS (pH 7.4) containing 1% bovine serum albumin, 10% normal goat serum, 0.3 M glycine and 0.1% Tween 20 for 1 h to block non-specific protein–protein interactions, and to obtain permeabilized cells. Then, cells were incubated with anti-NPRB antibody (5 μg/mL; Abcam) overnight at 4°C, whereas control experiments were done using isotype antibody (5 μg/mL; Abcam). The secondary antibody was goat anti-rabbit IgG (1/250 dilution, 1 h) and cells were stained with Hoechst 33342 (4 μg/mL, 10 min). Finally, cells were imaged and analyzed with a confocal laser scanning fluorescence microscope.

### Transport across the BBB

To assess the transport ability of CNP-modified lipid vesicles across the BBB, a BMVECs/U87-MG cell co-culture BBB model was established according to a previous report [47]. Briefly, the membrane of the upper insert (Corning, New York, NY, USA) was coated with gelatin (2%, *w/v*, D-Hank's buffer solution) for 1 h and BMVECs were seeded on the membrane at 4×10^4^ cells/insert. The culture medium was replaced every 2 days. After 6 days, U87-MG cells were seeded on the bottom of the lower well at 6×10^3^ cells/well and co-cultured for 24 h with BMVECs in the upper insert. Finally, the model was established and ready for experimentation. Different formulations (CNP-modified vinorelbine lipid vesicles, vinorelbine lipid vesicles, free vinorelbine) were added separately to the upper insert and the final concentration of vinorelbine was 100 nM. In contrast, a control group was incubated with a cGMP-dependent protein kinase inhibitor, Rp-8-CPT-cGMPS (100 μmol/L; Enzo Life Sciences, Geneva, Switzerland), for 1 h before addition of a drug formulation. After 3 h, the medium in the upper insert was replaced with a fresh BMVECs culture medium. After incubation for 48 h, survival of U87-MG cells in the lower well was determined by a SRB staining assay. The transport ability across the BBB was evaluated by the inhibitory effect in the lower insert of the BBB model.

### Uptake in HUVECs and brain glioma cells

Coumarin-labeled lipid vesicles were used to observe uptake by U87-MG cells and HUVECs. Cells were seeded in 12-well culture plates at 1.5×10^5^ cells/well in 2 mL of growth medium and cultured at 37°C. Afterwards, cells were incubated with free coumarin, coumarin lipid vesicles, or CNP-modified lipid vesicles at a final concentration of 1 μM coumarin for 3 h. Cells were washed with PBS (pH 7.4) thrice and resuspended in 300 μL PBS (pH 7.4) after filtration through a 400-mesh sieve. Finally, cellular uptake was measured using a FACScan flow cytometer (Becton Dickinson, San Jose, CA, USA) with 1×10^4^ events collected, and cellular uptake indicated by fluorescence intensity.

### Toxicity to brain glioma cells and HUVECs

To evaluate cytotoxic effects, U87-MG cells and HUVECs were seeded separately in 96-well culture plates at 5×10^3^ cells/well and grown in culture medium for 24 h. Then, cells were exposed to different formulations: free vinorelbine, vinorelbine lipid vesicles, or CNP-modified vinorelbine lipid vesicles. The final concentration of vinorelbine for U87-MG cells was 0–200 nM and for HUVECs was 0–400 nM. Blank culture medium was used as a blank control. After incubation for 48 h, cytotoxic effects were determined by a SRB staining assay based on measurement of absorbance at 540 nm using a microplate reader (Infinite F50; Tecan Group, Shanghai, China). Survival was calculated using the following formula: Survival % = (A_540 nm_ for treated cells/A_540 nm_ for control cells) × 100%, where A_540 nm_ is the absorbance value. Dose-effect curves were plotted from the data of triplicate assays.

### Apoptosis of brain glioma cells

Apoptosis of brain glioma cells induced by drug treatment was identified using a fluorescein annexin V staining kit (KeyGen Biotechnology, Beijing, China). Briefly, U87-MG cells were seeded at 5×10^5^ cells/well into six-well culture plates and incubated for 24 h at 37°C. Then, cells were exposed to free vinorelbine, vinorelbine lipid vesicles, or CNP-modified vinorelbine lipid vesicles for 6 h. The final concentration of vinorelbine for U87-MG cells was 50 nM. Blank culture medium was used as a blank control. After incubation for 12 h, cells were assessed by flow cytometry (Becton Dickinson) according to manufacturer instructions. Each assay was repeated in triplicate.

Expression of apoptotic enzymes and proteins (caspase 3, caspase 8, p53, survivin) in U87-MG cells was determined using high-content screening analyses. Briefly, U87-MG cells were cultured in 96-well plates for 24 h, followed by addition of various formulations at 50 nM vinorelbine, and blank culture medium was added as a blank control. After incubation for 6 h, cells were fixed using paraformaldehyde (4%, 15 min), permeabilized with 0.5% Triton X-100 in PBS (pH 7.4) for 15 min and blocked with PBS (pH 7.4) containing 10% goat serum and 0.3 M glycine for 2 h at room temperature. Then, cells were incubated with the primary antibody (1/250 dilution; Sangon Biotech, Shanghai, China) overnight at 4°C, followed by incubation with secondary antibody conjugated with Alexa Fluor®-488 (1/500 dilution; Beyotime, Beijing, China) for 2 h at room temperature. Cells were stained with Hoechst 33342 (2 μg/mL; Beyotime) for 10 min. Finally, the fluorescence intensity of cells was analyzed using the Operetta® high-content screening system (PerkinElmer, Waltham, MA, USA) and calculated with the Columbus system.

To evaluate ROS-related apoptotic signaling pathways, U87-MG cells were seeded in six-well plates at 5×10^5^ cells/well and incubated for 24 h. The formulations added were the same as those mentioned above, and were used to treat cells for 12 h at 37°C. After incubation for 12 h, cells were stained with Dichloro-dihydro-fluorescein diacetate (1 μM; Beyotime) for 10 min, followed by washing, harvesting and resuspension in PBS (pH 7.4). Finally, the fluorescence intensity of cells was determined immediately *via* flow cytometry.

### Disruption of neovasculatures *in vitro*

A three-dimensional Matrigel-based tube formation assay was used to assess the activity of drug formulations against the neovasculatures established with HUVECs. Briefly, a 96-well culture plate was coated with Matrigel (50 μL/well; BD Biosciences, Franklin Lakes, NJ, USA) for 30 min at 37°C. HUVECs (1×10^4^ cells/well) were resuspended with serum-free DMEM containing free vinorelbine, vinorelbine lipid vesicles, or CNP-modified vinorelbine lipid vesicles, and then loaded on top of the Matrigel. The final concentration of vinorelbine was 0.2 μM, and drug-free culture medium was used as the blank control. After incubation for 10 h at 37°C, tube formulation (capillary-like structures) was observed in three random visual fields using two-dimensional microscope images of the culture dish (Caikon Optical Instruments, Shanghai, China).

### Brain glioma-bearing mouse model

To evaluate drug distribution and overall anticancer efficacy *in vivo*, a brain glioma-bearing mouse model was established by intracranial implantation in male BALB/c nude mice (18–20 g). All procedures were carried out according to the *Guidelines of the Institutional Authority for Laboratory Animal Care of Peking University*. Briefly, nude mice were anesthetized with 4% chloral hydrate (10 μL/g) and fixed in a stereotactic device (RWD Life Sciences, Shenzhen, China). After an incision to expose the cranium, a burr hole was drilled 1.0-mm anterior from the coronal suture, 3.5 mm right-lateral from the sagittal suture, and 3 mm in depth. U87-MG cells (3×10^5^ cells/3 μL) were implanted into the inoculation point of each mouse at 1 μL/min.

### Imaging in brain glioma-bearing mice *in vivo*

Non-invasive optical imaging systems were used to observe the real-time distribution and tumor-accumulation ability of systemic DiR-labeled lipid vesicles in brain glioma-bearing nude mice. Fourteen days after intracranial implantation of U87-MG cells, mice were divided into four groups (three mice per group) and administered (*via* the tail vein) injections of free DiR, DiR lipid vesicles, or CNP-modified DiR lipid vesicles at 100 μg/kg for each mouse. Physiologic saline was injected as a blank control. Mice were imaged at 1, 3, 6, 12, 24 and 48 h using a Kodak multimodal imaging system (Carestream Health, Toronto, ON, Canada).

To further observe distribution of tumor masses and the major organs of glioma-bearing mice, the latter were killed at 48 h, followed by immediate removal of the brain, heart, liver, spleen, lungs, and kidneys. Fluorescence signal intensities in different tissues were photographed using a Kodak multimodal imaging system (Carestream Health).

### Disruption of neovasculatures and anticancer efficacy in brain glioma-bearing mice

After tumor inoculation, brain glioma-bearing mice were divided randomly into four groups of 11. At 12, 14 and 16 days, mice were treated with free vinorelbine, vinorelbine lipid vesicles, or CNP-TPGS-modified DiR lipid vesicles (*via* the tail vein) at 2.5 mg/kg vinorelbine. Mice in the blank control group were treated with physiologic saline instead.

To evaluate the disruptive effect on neovasculatures of brain glioma, three mice from each group were killed at 20 days to prepare frozen slices of brain tissue. These frozen slices were stained with the primary antibody rabbit (anti-CD31, 1/300 dilution; Abcam) and secondary antibody (Alexa Fluor 488-conjugated goat anti-rabbit IgG; 1/500 dilution; Beyotime) [48]. Then, slices were stained with 2 μg/mL Hoechst 33342 for 10 min. Finally, CLSM was undertaken to observe disruption of neovasculatures in brain glioma by measurement of fluorescence.

To evaluate the preliminary toxicity on various formulations, the blood indicators and histopathological changes were examined before and after sacrificing the mice, respectively. The blood analyses were conducted with an MEK-6318K Hematology Analyzer (Nihon Kohden, Japan). The histopathological observations were performed on the isolated heart, liver, spleen, lung and kidney tissues by hematoxylin-eosin stained paraffin slices.

The remaining eight mice in each group were used to monitor survival. Survival time was calculated from day 0 (tumor inoculation) to the day of death. Kaplan–Meier survival curves were plotted for each group.

### Statistical analyses

Data are the mean ± standard deviation (SD). ANOVA was used to determine the significance among groups, after which the Bonferroni correction was used for multiple comparisons between individual groups.

## CONCLUSIONS

In the present study, a novel type of CNP-modified vinorelbine lipid vesicles was developed. These vesicles could transport vinorelbine effectively across the BBB *via* NPRB-mediated transcytosis and opening of tight junctions. They could also disrupt brain glioma neovasculatures and eliminate brain glioma cells by targeting cellular uptake and then cytotoxic injury. In addition, CNP-modified vinorelbine lipid vesicles induced apoptosis of brain glioma cells. The apoptotic mechanisms were associated with the activations of caspase 8, caspase 3, p53, and ROS as well as inhibition of survivin. In conclusion, CNP-modified lipid vesicles could be used to treat brain glioma and to disable glioma neovasculatures.

## SUPPLEMENTARY MATERIALS FIGURE AND TABLE


