# Supplementary material for: C-type natriuretic peptide-modified lipid vesicles: fabrication and use for the treatment of brain glioma
Source: Oncotarget. 2017 Mar 29;8(25):40906–21. doi: 10.18632/oncotarget.16641 (PMC5522305; doi:10.18632/oncotarget.16641)
Supplement: Supplementary file 1 [file oncotarget-08-40906-s001.pdf]

C-type natriuretic peptide-modified lipid vesicles: fabrication and use for the treatment of brain glioma

SUPPLEMENTARY FIGURE AND TABLE

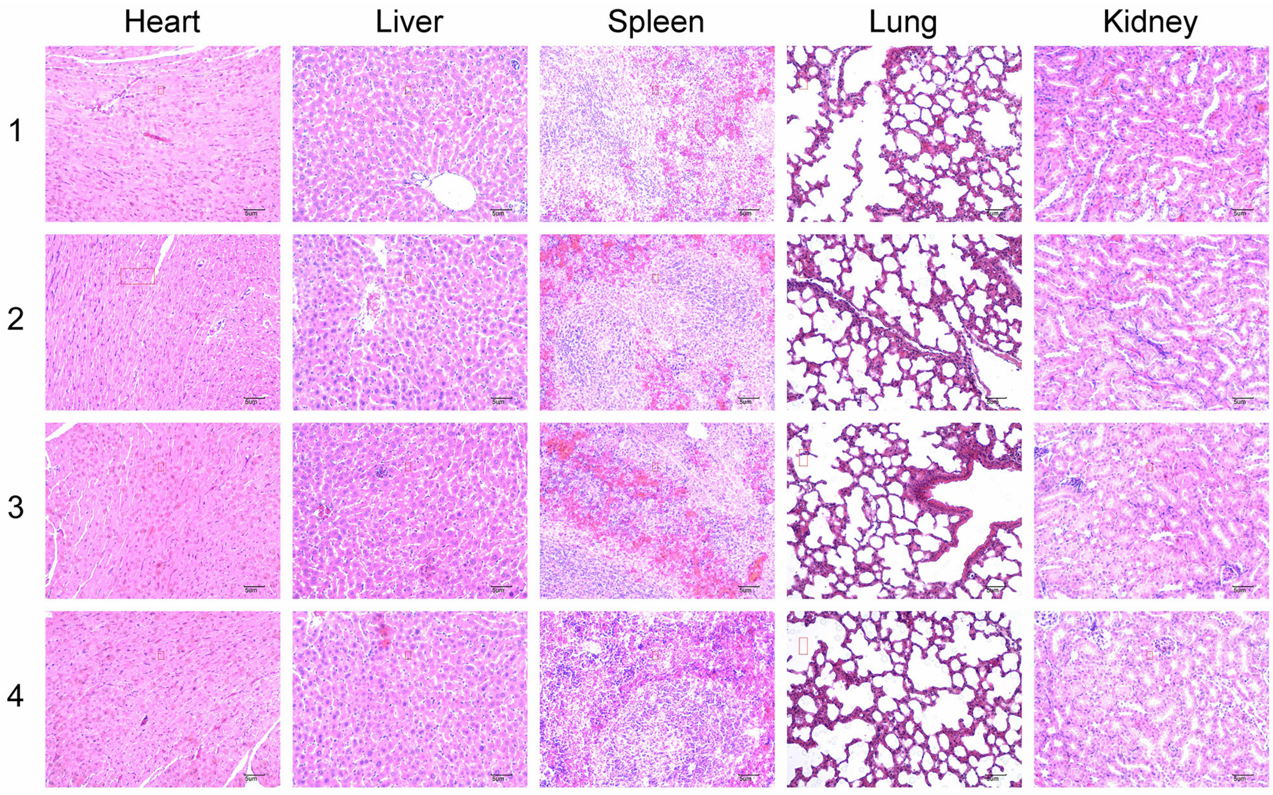

Supplementary Figure 1: Histopathological observations on the heart, kidney, liver, lung, and spleen tissues in glioma-bearing mice treated with various formulations (Bar = 5µm). Notes: 1, physiologic saline; 2, free vinorelbine; 3, vinorelbine lipid vesicles; 4, CNP-modified vinorelbine lipid vesicles.

Supplementary Table 1: Blood examination of glioma-bearing mice after treatment with various formulations at day 16 after inoculation

Assay	Physiological saline	Free vinorelbine	Vinorelbine lipid vesicles	CNP-modified vinorelbine lipid vesicles
WBC[a] (10^9/L)	7.80±1.32	11.00±0.85	6.20±1.82	10.50±2.42
RBC[b] (10^{12}/L)	8.73±0.29	8.40±0.16	8.36±0.42	8.55±0.34
HGB[c] (g/L)	137.67±10.50	136.00±8.19	137.00±9.54	137.00±1.73
MCV[d] (fL)	46.07±0.49	48.57±1.15*	45.83±1.06	47.63±0.23
HCT[e] (%)	40.20±0.95	40.83±1.71	38.30±2.80	40.70±1.55
MCH[f] (pg)	15.77±0.71	16.20±1.05	16.40±0.35	16.07±0.49
RDW[g] (%)	14.70±0.46	14.93±0.59	13.77±0.12	14.50±0.40
PCT[h] (%)	0.18±0.06	0.28±0.06	0.24±0.04	0.21±0.02
MPV[i] (fL)	3.63±0.15	3.97±0.38	3.73±0.12	3.73±0.15
PDW[j] (%)	13.03±0.50	13.47±0.12	13.33±0.21	13.17±0.40
MID[k] (10^9/L)	0.57±0.15	1.33±0.31	0.70±0.26	1.33±0.40
GRN[l] (%)	6.70±1.14	8.23±1.25	4.50±0.78	7.10±2.07

Notes: a, white blood cells; b, red blood cells; c, hemoglobin; d, mean corpuscular volume; e, hematocrit; f, mean corpuscular hemoglobin; g, red cell distribution width; h, plateletcrit; i, mean platelet volume; j, platelet distribution width; k, intermediate cell; l, neutrophile granulocyte. *$p < 0.05$, vs. physiological saline. Data are the mean ± standard deviation (n = 3).
